# Understanding Technology Preferences and Requirements for Health Information Technologies Designed to Improve and Maintain the Mental Health and Well-Being of Older Adults: Participatory Design Study

**DOI:** 10.2196/21461

**Published:** 2021-01-06

**Authors:** Haley M LaMonica, Tracey A Davenport, Anna E Roberts, Ian B Hickie

**Affiliations:** 1 Brain and Mind Centre The University of Sydney Camperdown Australia

**Keywords:** aging, mental health, technology, mobile phone, community-based participatory research, health care reform, stakeholder participation

## Abstract

**Background:**

Worldwide, the population is aging rapidly; therefore, there is a growing interest in strategies to support and maintain health and well-being in later life. Although familiarity with technology and digital literacy are increasing among this group, some older adults still lack confidence in their ability to use web-based technologies. In addition, age-related changes in cognition, vision, hearing, and perception may be barriers to adoption and highlight the need for digital tools developed specifically to meet the unique needs of older adults.

**Objective:**

The aim of this study is to understand the use of technology by older adults in general and identify the potential barriers to and facilitators of the adoption of health information technologies (HITs) to support the health and well-being of older adults to facilitate implementation and promote user uptake. In addition, this study aims to co-design and configure the InnoWell Platform, a digital tool designed to facilitate better outcomes for people seeking mental health services, to meet the needs of adults 50 years and older and their supportive others (eg, family members, caregivers) to ensure the accessibility, engagement, and appropriateness of the technology.

**Methods:**

Participants were adults 50 years and older and those who self-identified as a supportive other (eg, family member, caregiver). Participants were invited to participate in a 3-hour participatory design workshop using a variety of methods, including prompted discussion, creation of descriptive artifacts, and group-based development of user journeys.

**Results:**

Four participatory design workshops were conducted, including a total of 21 participants, each attending a single workshop. Technology use was prevalent, with a preference indicated for smartphones and computers. Factors facilitating the adoption of HITs included personalization of content and functionality to meet and be responsive to a consumer’s needs, access to up-to-date information from reputable sources, and integration with standard care practices to support the relationship with health professionals. Concerns regarding data privacy and security were the primary barriers to the use of technology to support mental health and well-being.

**Conclusions:**

Although HITs have the potential to improve access to cost-effective and low-intensity interventions at scale for improving and maintaining mental health and well-being, several strategies may improve the uptake and efficacy of technologies by the older adult community, including the use of co-design methodologies to ensure usability, acceptability, and appropriateness of the technology; support in using and understanding the clinical applications of the technology by a digital navigator; and ready availability of education and training materials.

## Introduction

### Strategies for Healthy Aging

The global population is aging rapidly. Within the next 40 years in Australia, for example, one-third of the population will be aged 50-65 years and a further quarter will be 65 years and older [1]. The economy will require a level of productivity from these people not previously seen. As such, there is a growing interest in strategies for supporting and maintaining health and well-being in later life to improve the social and economic participation of older adults to meet the demands of an aging society [2]. Efforts aimed at optimizing mental health and well-being are important contributors to achieving this mission.

### Internet Use Among Older Adults

The international literature indicates that approximately two-thirds of adults aged 65 years and older report internet use [3,4], and these older adults also represent the fastest-growing group of internet users [5]. Thus, using health information technologies (HITs) for mental health screening, intervention delivery, and routine outcome monitoring will be an increasingly viable option for older adults globally. The increase in internet use among this population has spurred a growing interest in the development and implementation of HITs for improved health and well-being for older adults [6-8].

### HITs

HITs are being developed rapidly for improving the delivery of mental health care for both consumers and health professionals and for facilitating improved self-management of care [9]. To that end, HITs have been shown to be effective for the management and treatment of symptoms in a range of mental health and medical conditions, including depression [10-12], diabetes [13], weight loss [14], problematic alcohol use [15], sleep [16], exercise [17], and social connectedness [18]. Barring some exceptions, including a diet diary app for older adults with age-related macular degeneration [19], few HITs have been designed specifically with older adults in mind. As such, this represents a largely untapped market for potential web-based tools to improve the health and well-being of older adults.

Importantly, in a study of older adults (N=221) presenting to a specialized memory clinic for concerns regarding new-onset cognitive decline and/or mood symptoms, most participants (198/209, 94.6%) reported that they would find it useful to be able to access a website designed to support healthy aging, including physical health and cognition, self-management of existing conditions, and routine tracking of changes in health outcomes over time. Similarly, most respondents also reported interest in a website designed to specifically measure mood-related concerns and changes (172/206, 83.5%) [20]. Despite having interest in and motivation to use HITs to improve their health and well-being [20,21], some older adults still lack confidence in their ability to use web-based technologies [22,23]. It has been demonstrated that older adults will only adopt new technologies when their apparent usefulness and usability outweigh concerns related to technological complexity and decreased social connection [24]. In light of these factors and age-related changes in cognition, vision, hearing, and perception, it is critical that HITs be tailored to the older adult community, taking into consideration their unique needs as users.

### Participatory Design

Using strategies to enhance community and consumer acceptability, usability and engagement with HITs is a priority in the health, medical, and research sectors internationally [25,26]. To this end, co-design methodologies, including participatory design and user testing, are widely recognized as key to ensuring the quality, usability, and acceptability of HITs for specific user groups—in this case, older adults. Research has shown that the active participation of all stakeholders throughout the design of technical systems and services helps ensure that the end product meets the needs of its intended user base, improves usability, and increases engagement of all individuals [27-29]. Importantly, there is an emerging evidence base reflecting the benefits of co-design with older adults, including those with dementia, and their family and caregivers, to enable strengths-based, person-centered care [30,31]. Our research team’s established co-design methodologies explicitly position users as empowered participants in all stages from design and development through to implementation and impact evaluation [28,29,32,33].

### The InnoWell Platform

In 2017, the Australian Government Department of Health and InnoWell Pty Ltd (a joint venture between the University of Sydney and PwC, Australia) entered into a 3-year funding agreement to deliver Project Synergy (2017-2020), a series of collaborative research trials with the specific purpose of co-designing and implementing innovative HIT solutions, including the InnoWell Platform, to enable improved mental health service delivery in Australia, facilitating better outcomes for people with lived experience, supportive others, health professionals, and service providers [9]. As described in detail by Davenport et al [34], the co-designed InnoWell Platform was developed through Project Synergy (by InnoWell) to collect information from multiple sources to formulate a comprehensive understanding of a consumer’s needs and to monitor their progress over time. These sources comprise web-based, self-reported questionnaires assessing a range of health domains (ie, psychological distress, suicidal thoughts and/or behaviors, daily functioning, depressed mood, cognition, sleep-wake cycle, social connectedness) from both consumers and their health professionals and objective behavioral data collected via third-party integrations (eg, Fitbit trackers). The multifaceted and multidimensional assessment results are designed to be understandable directly by consumers and to be reviewed in collaboration with their health professional to promote shared decision making and collaborative care and to facilitate routine outcome monitoring, clinical review, and coordinated care to ensure that all consumers receive the right care first time.

### Objectives

We now aim to customize and configure the InnoWell Platform to meet the needs of an older age group (50 years and older) and their supportive others (eg, family members, caregivers) to ensure the accessibility, engagement, and appropriateness of the technology. We defined *older adults* as those aged 50 years and older as this aligns with our own previous work investigating technology use and preferences among this group [20]. Furthermore, the age of 50 years relates to the onset of disorders in later life [35] and the identified age range during which it is recommended to address risk factors (ie, cardiovascular disease, obesity, diabetes) known to undermine healthy aging [36]. Furthermore, we seek to understand the potential barriers and facilitators of HIT use for older adults to better identify and understand ways to promote adoption and facilitate successful implementation.

## Methods

### Participants

This study aimed to recruit up to 50 participants, including a combination of older adults and their supportive others. The inclusion criteria for participation in the study required participants to be aged 50 years and older or self-identify as a supportive other (eg, family member, caregiver), to be proficient in English, and to have completed the required informed consent process.

### Recruitment Strategies

This study was advertised through the University of Sydney’s Brain and Mind Centre, including through active research clinics working with older adults and via nongovernmental (ie, Dementia Australia) and private organizations (ie, InnoWell) associated with the Brain and Mind Centre. The recruitment strategy included the use of postcards and A3/A4 posters in both paper-based and digital forms, depending on the preference of the advertising site (eg, poster displays, postcards at reception, posting the digital advertisements on the research sites and social media pages).

To avoid any perceived coercion, recruitment was passive such that a potential participant needed to contact the senior research health professional (HL) who, only on a potential participant’s request, then forwarded the study Participant Information Sheet and Consent Form. All participants were provided with detailed information about the study both before attending a participatory design workshop and again on arrival at the workshop. At the beginning of each workshop, the facilitators provided the participants with an opportunity to ask questions and clarify details of the research before providing written informed consent. Potential participants were reminded that participation was entirely voluntary and that if they agreed to participate, they could withdraw their consent at any time without being required to provide any reasons and with no impact on their relationship with the University of Sydney, the Brain and Mind Centre, InnoWell, or the participating clinics through which they were recruited.

### Participatory Design Workshops

A series of 4 group-based workshops of approximately 3-hour duration, each with up to 10 participants, were conducted with older adult stakeholders to discover, evaluate, and prototype acceptable design solutions for the InnoWell Platform. These sessions involved an iterative knowledge translation process so that initially generated ideas can be further developed (and fed back on) by participants in subsequent workshops (Multimedia Appendix 1 presents a sample agenda). All workshops were coordinated by 2 facilitators (one of whom was a health professional) and a scribe was present to take detailed notes. Two facilitators were considered important; the first facilitator guided the research questions and session plan, and the second facilitator ensured that all participants’ voices were heard within the workshop.

As in our previous co-design research [29], the facilitators used a variety of methods within the workshops, including prompted discussion, prototyping, creation of descriptive artifacts, and group-based development of user journeys (a series of steps illustrating how an individual might interact with the prototype). It is important to note that the InnoWell Platform is being designed and developed iteratively; therefore, although a version of the InnoWell Platform has been built, the participants were not exposed to the technology as part of the workshops to avoid bias in their thinking. As such, a *blue sky* approach (ie, brainstorming without limitations or practical constraints) was used for prototyping to ensure that the necessary features and functionality that may be unique to the older adult community were captured. On the basis of previous studies exploring the use of technology for health-related purposes by older adults [4,24,37,38], a number of critical areas were explored, including (1) preferred devices, (2) common uses of technology, (3) use of technology to support health and well-being, (4) features or functionality that promote user engagement, (5) interest in and preferences for digital health services, and (6) concerns related to data privacy and confidentiality.

### Data Analysis

Interpretation of the qualitative data from the workshops, including scribe notes and artifacts, followed established thematic techniques [39]. All raw data were reviewed and checked across all participants by a senior research health professional (HL), and a coding framework outlining all key concepts was developed. Data were coded in NVivo 12 software (QSR International) using this framework. The coding followed an established iterative process of reading, coding, and exploring the pattern and content of coded data, followed by reflection and discussion. Similarities and differences in opinion were examined, and differences were dealt with through discussion to reach consensus. Coding was conducted initially by the senior research health professional (HL) and a randomly selected subsample of 10% was checked for inter-rater reliability by a research officer (AR); agreement was substantial (κ=0.631) [40]. In alignment with the topics explored in the participatory design workshops, themes were then organized as follows: (1) preferred device; (2) well-being as a concept; (3) barriers to and facilitators of technology use generally; and (4) barriers to and facilitators of technology use to support mental health and well-being, including a prototype of the InnoWell Platform configured for older adults. All themes were checked against each other and back to the original data to ensure that all relevant references had been collated. This process resulted in a thematic framework that was internally coherent and consistent.

### Ethics

This research study was approved by the University of Sydney’s Human Research Ethics Committee (Project No. 2019/172).

## Results

### Demographics

A total of 4 participatory design workshops (all 3 hours in duration) were held between September and November 2019. The aim of each workshop was to actively engage the older adult community in discussions about how technology may be used to promote mental health and maintain well-being. A total of 21 adults (43% female) aged 50 years and older attended the workshops, 2 of whom also identified as supportive others. All participants attended only one workshop. Although the sample size was smaller than planned, the richness of the data and the consistency of the themes indicated that we had reached saturation. To ensure participant confidentiality, further demographic details were not collected as part of this study. No participants expressed concern about or experienced any distress in any of the workshops.

### Technology Preferences

When asked *What is your favorite piece of technology*, participants reported a range of preferences, including *computers, tablets, eBook readers, basic mobile phones, wearables, and televisions.* However, the *smartphone* was the most frequently referenced device (Textbox 1) for several reported reasons:

my phone is always on...used for a lot of functional things—news, transport, a lot of informational things.

Workshop 1

I read the paper on my phone.

Workshop 2

...to stay connected.

Workshop 4

I use notes a lot for writing poetry.

Workshop 4

Computers were also referenced frequently as a preferred device because of the diversity of available functionality, such as * “creative work...music...Photoshop,” “YouTube extreme sports...puts you in places you’ve never been...online shopping,” “use it for music composition and practice,”* and *“love using YouTube...added value for my work” *(all from Workshop 1).

*Tablets* were largely referenced in relation to games and ease of access to information (ie, news, politics, sports). However, there was an indication that *smartphones, tablets*, and *computers* were used interchangeably for the purpose of accessing the internet, with 1 participant noting:

It’s all the same to me...if I’m out it’s the phone, at home it’s the tablet or phone.

Workshop 4

Participants also referenced the use of apps and e-tools both in relation to entertainment, for example, *Spotify for* “access to music…listen to podcasts” (Workshop 1) and to support health and well-being, including *“Headspace app for meditation...keeping in contact with kids through various apps”* (Workshop 3); “Lumosity…I had to wean myself off it…I was becoming competitive with it and couldn’t get to sleep” (Workshop 3); and “family history and that’s a real brain teaser to follow different leads…it’s very complex and good for the brain” (Workshop 3).

**Codes related to technology preferences theme (63 references).**

Preferred devices used by older adults include:

- Smartphone (18 references)
- Computer (15 references)
- Apps and e-tools (10 references)
- Tablet (9 references)
- Basic mobile phone (3 references)
- eBook readers (3 references)
- Wearables (3 references)
- Television (2 references)

### Well-Being

As shown in Textbox 2, two primary themes emerged from the discussions about well-being, with *concepts* being referenced more frequently than *strategies.* In relation to the former, references to *health and functional capacity* were the most common. Participants consistently characterized well-being as a holistic combination of mental and physical health, with one stating:

It’s not just the absence of sickness but capacity to do what you want with your body, such as reach maximum heart rate…the presence of strength…feeling good.

Workshop 3

There were also several references to *self-awareness and acceptance,* noting that well-being relates to *“relationship to yourself or with others”* (Workshop 1) *and “my own state of mind”* (Workshop 2*); well-being* was conceptualized as being *“personal to you...for example, someone immobile for life could still have well-being”* (Workshop 3)*.*

Although referenced less frequently, participants indicated that well-being also relates to *safety* (ie, *“feeling good and feeling safe”* [Workshop 3])*, social connectedness* (ie, *“connection to people”* [Workshop 1])*, and resilience or the ability to “move through” (Workshop 3) challenging events.* Although there was consensus as to the conceptualization of well-being, some participants indicated that the term had become a *buzz word* *(Workshop 2)* used for *marketing purposes.* In addition, it was noted that well-being can be negatively impacted by *stigma,* as 1 participant stated:

Those who struggle most with stigma are those with mental health issues...frustrated by telling their story over and over.

Workshop 3

Several strategies to promote or maintain well-being were referenced with similar frequency, including *leisure activities*, such as *a break from work (Workshop 2)* and *a massage (Workshop 3)* and *diet and exercise (Workshop 2)*. Although some participants referenced the importance of *social connectedness (Workshop 2)*, others indicated a need for *self-reliance*, noting:

I’d manage it myself, wouldn’t want to burden other people.

Workshop 2

Finally, there were mixed responses regarding the value of *information and tips*, with 1 participant noting:

A friend who worked in arthritis research used to send me information and I trusted it.

Workshop 2

Another stated:

I wouldn’t be interested in daily tips.

Workshop 2

**Codes related to well-being theme (48 references).**

Concepts (37 references)

- Health and functional capacity (15 references)
- Self-awareness and acceptance (10 references)
- Marketing purposes (3 references)
- Social connectedness (3 references)
- Resilience (2 references)
- Safety (2 references)

Strategies (11 references)

- Leisure activities (3 references)
- Diet and exercise (2 references)
- Information and tips (2 references)
- Self-reliance (2 references)
- Social connectedness (2 references)

### Barriers to and Facilitators of Technology Use

When discussing the use and impact of technology in daily life, two primary themes emerged—*barriers and facilitators* (Textbox 3), with the latter being referenced with greater frequency. In particular, *social connectedness* was one of the primary ways in which participants were making use of technology, with participants commenting:

Technology is my communication...email and text are important for me to keep in touch.

Workshop 2

It’s a huge difference to me with three children who live in the US.

Workshop 3

Skype/FaceTime with family makes you feel connected.

Workshop 3

It creates easier, less formal contact with friends.

Workshop 3

Interestingly, the potential for technology to drive *social disconnection and miscommunication* was noted as a potential barrier as a participant stated:

It worries me that young people don’t know a life without a screen…they don’t know how to connect without an app.

Workshop 3

One participant questioned:

Why don’t you just call...there can be miscommunication with texting.

Workshop 1

Furthermore, it was also agreed that connecting via technology is *not equivalent to in-person*. One participant commented:

I know I can do it on the computer, but I enjoy the contact...it’s having a human element.

Workshop 3

Another stated:

If I can do it as a video, then I can see my grandchildren...but I can’t have a hug.

Workshop 3

*Games*, such as “Word with Friends...Candy Crush…Bridge” (*Workshop 4*) and “Spider Solitaire” (Workshop 4) and *information and new learning* were also frequent uses of technology, including references to websites related to news and travel and “YouTube…gives you video tutorials” (*Workshop 1*). Although participants indicated that the *ease of access* to information was a facilitator to technology use, noting, “You can’t beat Wikipedia for instant information about anything” (*Workshop 1*), it was also highlighted that this *changes the way we think*. For example, in relation to the consumption of media, one participant stated:

The way I read and get information now is much more snapshot rather than long form journalism.

Workshop 1

Another participant noted:

Problem-solving is lacking as you can just tap on a screen and get information...they don’t question whether it’s the right information.

Workshop 3

In relation to the latter, participants indicated that the *credibility of the source* was important when using the internet or web-based tools. Information produced by the government, reputable health organizations, universities and academics, and individuals with higher degrees or qualifications were more likely to be perceived as trustworthy and reliable. At the same time, however, the potential for *miscommunication and limited detail* was referenced, with some skepticism expressed about news sources, including:

We’re in the age of misinformation, they don’t want us to know the truth.

Workshop 1

If you can get someone to click on a headline, that’s more valuable than relating it to the article.

Workshop 1

In addition, 1 participant referenced *security concerns* in relation to web-based data sharing, stating:

Anything on the Internet I just don’t really trust, I don’t want to put my information of any kind out there.

Workshop 2

The potential *anxiety-provoking* nature of technology was referenced, albeit infrequently, with a participant commenting:

My older friends get anxious if something goes wrong and they don’t know how to fix it...they aren’t feeling confident.

Workshop 3

Digital literacy was characterized as a skill:

You have to learn it, like anything else.

Workshop 3

Finally, w*ork requirements* were noted as a driver of technology use, whereas *lack of interest* was a barrier.

**Codes related to barriers and facilitators to technology use theme (63 references).**

Barriers (28 references)

- Changes the way we think (9 references)
- Misinformation and limited detail (8 references)
- Social disconnection and miscommunication (4 references)
- Not equivalent to in-person (3 references)
- Anxiety (2 references)
- Lack of interest (1 reference)
- Security concerns (1 reference)

Facilitators (35 references)

- Social connectedness (13 references)
- Games (6 references)
- Information and new learning (6 references)
- Credibility of the source (4 references)
- Ease of access (4 references)
- Work requirements (2 references)

### HIT Use

As shown in Textbox 4, *barriers* and *facilitators* again emerged as the primary themes when discussing the use of technology specifically for health-related purposes and co-designing a prototype of the InnoWell Platform for older adults. Access to *information* was the primary facilitator referenced, in relation to being able either to *read up on a problem and present that to doctors* *(Workshop 1)* or *to go back to the internet to take the time to review and be critical of information* discussed in an appointment with a health professional *(Workshop 1).* Although HITs are *not equivalent to in-person care*, that is, “Need face-to-face to establish trust for subsequent phone or emails…you feel you know the person” *(Workshop 1)*, it was noted that they provide improved *access to care*. For example, the convenience, that is, “face-to-face is best, but over the phone is convenient” *(Workshop 1)* and anonymity afforded by technology may “be a good thing for people suffering mental health…if it was face-to-face or someone they knew they would be less likely to do it at all” *(Workshop 3).* Whether the technology was *endorsed by a health professional* or *endorsed by a family member or friend* were noted facilitators of HIT adoption and engagement, with participants indicating they would use it if “recommended…by my doctor” *(Workshop 2)* or “a certain friend or family member” *(Workshop 2)*.

It was recognized that the ability for technologies to be *integrated with health care* increases the transparency in care:

...the doctor used to just have it, now you have it on your computer...you have all your health information at your fingertips.

Workshop 4

In addition, it affords the opportunity for *coordinated care,* allowing shared information between treating health professionals. One participant stated:

I see it as a total package, it’s just part of your wellbeing. My different medical people should know if I have a heart issue or if I’m seeing a psychologist.

Workshop 2

As a caveat, *ownership and personal choice with data* provides consumers with the option “to decide who can see what and which doctors have access” *(Workshop 4)*. *Security concerns* were referenced as the primary barrier to HIT use, with concerns noted to include “once your email is connected, essentially hackers could get a lot” *(Workshop 4)*, “not getting jobs…not getting insurance” *(Workshop 3)*, and “people will target you with products based on information gathered” *(Workshop 3)*.

From a technical perspective, *user experience and customization* were referenced as facilitators of use. *Data tracking* was also an attractive feature, including the ability to track physical activity and weight loss using apps such as MyFitnessPal and via wearables (ie*, Fitbit).* In addition, *competition* is a potential driver for technology use. For example, 1 participant commented:

...subscribed to Lumosity for about a year...it was lots of fun...tried to improve my score to be in the top percentile for my age group.

Workshop 2

However, the *potential for misuse* was also identified as “over-notifications could feel like bullying” *(Workshop 1)* or result in an “obsession with the data” by users *(Workshop 1)*. *Data entry and tracking requirements* were also viewed as a potential barrier to use, as it “might be another stressor for some people” *(Workshop 3)*, particularly “once they are unwell” *(Workshop 3)*.

Although not raised as a personal concern by any of the participants, limitations in *digital literacy* was referenced as a potential barrier as it was noted that:

...technology is not usable by a lot people in my generation...at the moment there is a generational cut-off.

Workshop 4

In addition, *lack of interest* in HITs, including how they work, may also reduce uptake.

**Codes related to technology use to support health and well-being theme (308 references).**

Barriers (57 references)

- Security concerns (18 references)
- Lacks credibility of health professional (10 references)
- Not equivalent to in-person care (10 references)
- Potential for data misuse (9 references)
- Data entry and tracking requirements (8 references)
- Lack of interest (7 references)
- Digital literacy (1 reference)

Facilitators (59 references)

- Information (13 references)
- Data tracking (10 references)
- Access to care (8 references)
- Endorsed by health professionals (7 references)
- Coordinated care (5 references)
- Integrated with health care (5 references)
- User experience and customization (3 references)
- Ownership and personal choice with data (3 references)
- Competition (3 references)
- Endorsed by a family member or a friend (2 references)

Prototype features and functions for a digital platform customized for older adults (192 references)

- Barriers (34 references)
  Impersonal and social disconnection (5 references)
  Lacks credibility of health professional (5 references)
  Privacy and security risks (5 references)
  Competition (4 references)
  Limitations and potential errors (4 references)
  Anxiety about seeking help (2 references)
  Digital literacy (2 references)
  Generic information (2 references)
  Misinterpretation of information (2 references)
  Requirements for use (2 references)
- Facilitators (158 references)
  Personalization (32 references)
  Information and resources (15 references)
  Interoperability and data tracking (13 references)
  Credible source or endorsed by health professional (12 references)
  Interaction with health system (12 references)
  Prevention and risk reduction (11 references)
  Access (10 references)
  Recommendations and interventions (10 references)
  Anonymity (8 references)
  Goal setting (6 references)
  Personal data record (6 references)
  Education and training (5 references)
  Empowering (5 references)
  Social connection and support (5 references)
  Diagnosis (2 references)
  Supportive other functionality (2 references)
  User experience and design (2 references)

### Prototyping the InnoWell Platform for Older Adults

Building on the foundation of their experiences with health care systems and technology generally and HITs specifically, participants co-designed a prototype of the InnoWell Platform for older adults, identifying features and functionality that would be *barriers* to and *facilitators* of adoption and implementation. Importantly, facilitators were referenced far more frequently than barriers, potentially reflecting the interest in and increasing use of technologies to support health and well-being. The primary driver for use was *personalization* (ie, a tool that is designed to meet a consumer’s requirements and that responds to the data entered by a consumer to meet a consumer’s needs) as participants noted:

I can see a clear use...someone wakes up and feels bad or experiences a new symptom…they go on this software and it give them some triage to start with.

Workshop 1

...questions about you and your situation...sends you off to areas of the site that could be useful.

Workshop 3

It could be like a referral service to send you in the right direction.

Workshop 3

*Interoperability* (ie, the ability of the digital tool to exchange information with other technologies such as apps and wearables) and *data tracking* were identified as factors that may facilitate *personalization*. For example, one participant stated they would:

be able to input data about my arthritis, pain levels, tracking what’s happening with my fingers...I assume it would give me lots of information, things to do...take some ownership of my tracking.

Workshop 2

Interestingly, the need for personalization was coupled with a desire for *anonymity*. It was suggested that the user “could create a username” *(Workshop 1)*, with participants agreeing that “the information going in is so sensitive that I would only do it anonymously” *(Workshop 1)*. Furthermore, the ability to store health information in a *personal data record*, that is, “it knows all my background” (*Workshop 3*) was valued by participants. However, it is important to note that *privacy and security risks* were frequently referenced barriers as cybersecurity was characterized as “an arms race...people employed in security are constantly trying to stay in front of the hackers” *(Workshop 1)*.

The ability to find up to date *information and resources* was also referenced as a facilitator of use—a place to ask “those silly questions you just can’t ask in an intimidating environment” *(Workshop 2)* and gain better understanding of “what to tell your [general practitioner] GP…teaching you the things you need to tell your specialist” *(Workshop 2)*. However, there was some concern that the *misinterpretation of information* might be a barrier, recognizing a “risk of creating a device that leads people to self-diagnosing” *(Workshop 2)* and that *generic information* may not have much benefit if it is “not personalised” *(Workshop 3)* and “only answers a silly little thing” *(Workshop 3)*.

**Figure 1:**
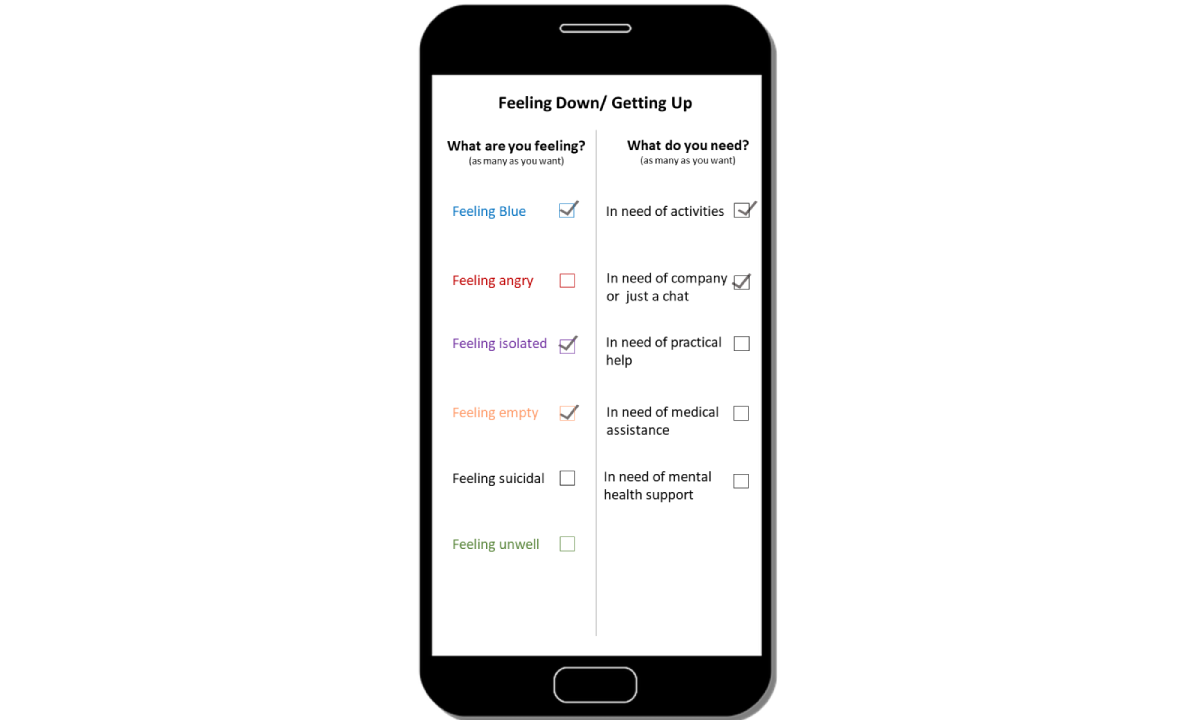
App prototype of a digital tool to support health and well-being.

Participants wanted there to be an *interaction with health care*, potentially as a “referral to a specialist” *(Workshop 1)*, a way to “fast track the system…direct you to service” *(Workshop 1),* or a tool to enhance the care provided by a health professional, that is, “If I brought it in and showed it to her, she’d probably work collaboratively with me.” *[Workshop 2*]*)*. *Recommendations and interventions* were also a desired feature, with ideas including “interventions to do balance exercises” *(Workshop 1)*, “video training about how to do a guided [meditation] session” *(Workshop 3)*, and “virtual group sessions” *(Workshop 3)*. However, the *potential for error* was cited as it was recognized that there is an inherent “risk with assuming that feeding information in means the outcomes will be right” *(Workshop 1)*. Figure 1 reflects one participant’s conceptualization of how he or she might use this type of tool. By inputting information about current symptoms and desired services or activities, the digital tool would then provide tailored recommendations.

**Figure 2:**
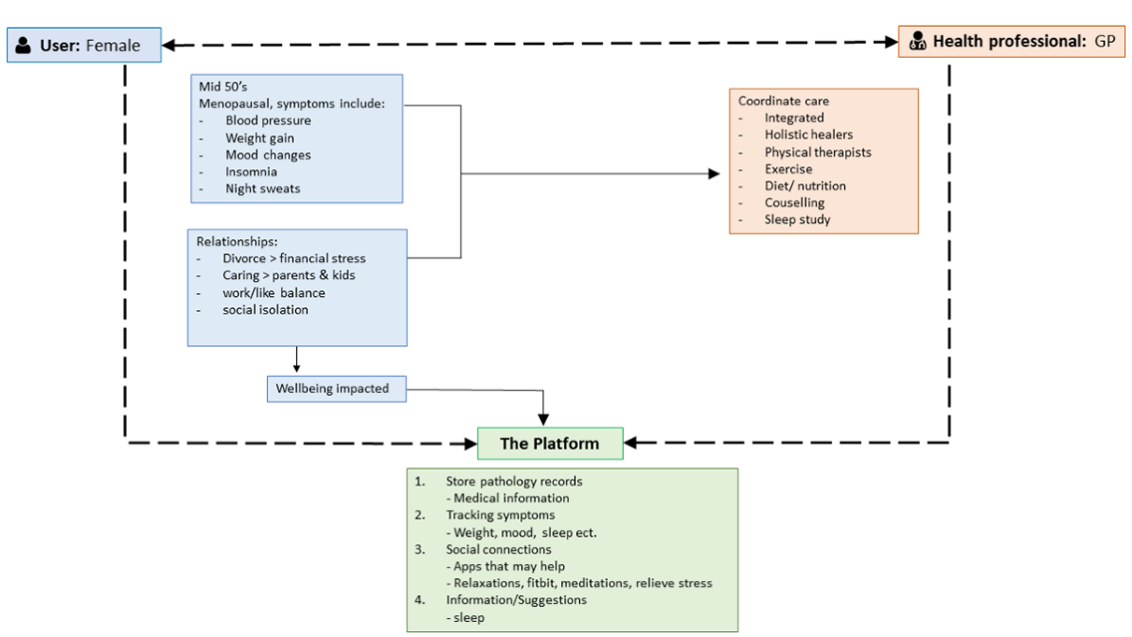
User journey demonstrating how a consumer might engage with the prototype.

Participants indicated that they would be more likely to use this type of tool if it came from *a credible source or was endorsed by health professionals*, highlighting the need for the content to be “developed by an organisation that is already trusted” *(Workshop 2).* Similarly, a digital tool was viewed to *lack the credibility of a health professional*, thereby potentially preventing use as participants were not interested in “replacing GPs or specialists” *(Workshop 2).* The potential for a digital tool to be *impersonal* was also referenced as a barrier, particularly for older adults who “could be more isolated…need someone that cares…the connection is still important” *(Workshop 1)*. Figure 2 presents a hypothesized user journey created by participants, highlighting the way in which they would use the prototype of the digital tool, including the information that they would input to personalize the results, the manner in which a health professional could make use of that information to coordinate care, and the support provided via the digital tool.

Although there was minimal reference to the need for the tool to be able to provide a user with a *diagnosis*, participants reported an interest in *prevention and risk reduction* or “something that would keep me away from the doctor…preventative but also health generative…keeps you healthy and active” *(Workshop 1)*. *Goal setting* was a potential motivator of use, with participants noting that users “would establish goals after going through a level of entering information” *(Workshop 1)* and recognizing that “goals could change as you go through” *(Workshop 1)*. On the other hand, *competition* was referenced as “demotivating…don’t want people to fail and say I won’t look at that again” *(Workshop 1)* and “a huge problem…a goal isn’t a goal unless there’s a success or failure measured” (*Workshop 1)*.

It was widely recognized that a digital tool has the potential to improve *access* to health care, particularly for individuals in remote areas where “distance becomes a disability” *(Workshop 1)*, helping “people not feel isolated when they are physically isolated” *(Workshop 2)*. *Empowering* users was also referenced as an important component, with a need for a strength-based approach because “older people are told you can’t do that anymore…celebrate what they are doing” *(Workshop 1).* Although referenced infrequently, participants indicated *that they were more likely to use a digital tool that had a good user experience and design.* Furthermore, it was recommended that users would benefit from *education and training* resources, “information on how to use it – a tutorial or mind map…give people an idea of what kind of help they could get” *(Workshop 3)*. This may be particularly relevant for older adults with poor *digital literacy* as “people will always be left out if it is on a computer” *(Workshop 1)* or for those who experience *anxiety related to help seeking,* where the technology should not be “too daunting” *(Workshop 1).* However, neither of these barriers were reported to be personal concerns for the participants.

## Discussion

### Technology Use by Older Adults

Our results align with previous findings that older adults routinely engage with a range of technologies, including computers, mobile phones, eBook readers, and tablets. Importantly, the preference for smartphones highlights a change in device use among older adults. Although previous studies have found that the use of home computers was nearly universal in a sample of older adults (93%), the use of smartphones was far less frequent (52%) [20]. The present results emphasize the rapid growth in smartphone ownership among older adults in recent years and the effect of aging [41], with younger people with greater digital literacy and experience with technology moving into the older age group. For example, among Americans aged 65 years and older, ownership of smartphones increased from 23% in 2013 to 42% in 2016 [4]. It is important to recognize that device adoption varies considerably with age, education, and household income. The proportion of older adults who own a smartphone or tablet is markedly higher for those aged 65 to 69 years (59% and 41%, respectively) relative to those aged 75 to 79 years (31% and 28%, respectively) [4]. Similarly, individuals with higher levels of education (a bachelor’s degree or beyond) are significantly more likely to have a computer and/or smartphone [20]. As such, consideration of the *digital divide* is crucial when considering technology use among older adults, as there may be barriers to access (eg, internet, smartphone) that preclude their engagement with web-based tools, including for, but not limited to, information and entertainment purposes and for the purposes of improving and maintaining health and well-being.

### Components of and Strategies to Support Well-Being

Participants generally conceptualized well-being as an absence of illness (ie, *feeling healthy*) and the capacity to fulfill one’s goals and carry out activities of one’s choice. However, there was also recognition that illness or disability and well-being are not mutually exclusive; rather, there is a need for personal awareness and acceptance of self. The fulfillment of basic life needs, namely, safety in one’s surroundings, connection to others, and resilience (ie, the ability to bounce back in the face of stressful events) were also referenced. Importantly, participants noted that stigma can detract from well-being as it may be a barrier to seeking and accessing help for mental health problems. The factors of well-being identified by participants align with the 6 components of the Ryff model of psychological well-being [42], which includes self-acceptance, mastery of the environment, autonomy, positive relationships, personal growth, and life purpose. Our results highlight the consistency of views on psychological well-being among older adults over more than 30 years and, importantly, suggest these are key targets for health-related interventions.

Several strategies to promote and maintain well-being were referenced equally, including maintaining a healthy diet and exercising regularly, engaging in leisure activities, and making use of health-related information and tips. Consistent with the recognition that social connectedness is a key determinant of health [43,44], participants agreed that social connectedness is an important component of well-being. However, this was balanced with a need to be self-reliant in maintaining one’s well-being so as not to burden others. HITs have the potential to provide low-cost intervention and prevention tools that are designed specifically to target components of well-being [42] and symptoms of mental illness, such as anxiety, depression, and problematic health behaviors (eg, alcohol, gambling, and smoking). In fact, a meta-analysis found that apps were superior to control conditions in improving stress levels and quality of life and depressive and generalized anxiety symptoms, with no marked difference relative to active interventions, including in-person treatment [45].

### Benefits and Pitfalls of Technology Use

Although there is no doubt that technology has the potential to facilitate social connection via phone calls, videoconferencing, text messaging, group chats, and even games, participants agreed that in-person connection remains a vital part of personal relationships and interactions with health professionals. In a recent study, it was shown that when allowed to rely on a smartphone for information, participants were less likely to speak to other people and felt less socially connected than those who were not allowed to use phones [46]. As evidenced by our participants, older adults will engage with social media to stay connected to family and friends; however, previous qualitative work in this area revealed that older adults prefer deep, thoughtful, one-on-one communications as opposed to the light-touch, group-based interactions promoted through social media [47]. These findings suggest that it is important to ensure that HITs are well integrated with the health care system, enabling the therapeutic relationship between a consumer and health professional as opposed to rendering it unnecessary. This may be a particularly important consideration for older adults who tend to experience greater degrees of social isolation and loneliness, which are known risk factors for health problems, including cognitive decline and depression [48].

In addition to social connection, several participants also reported engaging with technology to play games, such as *Candy Crush* and *Words with Friends*. Interestingly, older adults aged 65 years and older are the fastest-growing segment of new digital game players in Australia [49]. Although utility as a leisure activity is important, gaming may also present an opportunity for incidental cognitive assessment, where changes in game behavior or performance may be indicative of decline at its earliest stage, thereby facilitating early intervention strategies to mitigate known modifiable risk factors such as depression, midlife hypertension, midlife obesity, and low physical activity [50]. Importantly, a systematic review of gamified cognitive assessment and training paradigms found evidence suggestive of associated improvements in engagement, intrinsic motivation, and training outcomes (when relevant) [51,52]. In addition to promoting repeated engagement, gamifying cognitive tasks can improve usability, decrease test anxiety, and increase ecological validity [51]. Further research is now required to validate the application of game design principles to cognitive assessment to improve sensitivity to the earliest signs of decline and to cognitive training to promote engagement, real-world transfer, and sustainability of outcomes.

### The Potential Impact of Health Information Technologies

The disruption caused by the COVID-19 global pandemic has resulted in a greater need for and reliance on digital health care for screening, treatment, and ongoing maintenance of health. To this end, HITs offer a viable alternative for those who prefer or are required to use digital health care due to health concerns (eg, during the COVID-19 pandemic) and geographic, transport, or mobility constraints. One of the marked discrepancies between our study and others that have investigated technology use for health-related purposes by older adults relates to familiarity with and confidence in using technology. Although this was not a personal concern among our participants, a lack of familiarity with technologies has frequently been cited as a potential barrier to adoption for older adults [53], specifically in relation to web-based health care information seeking [54]. As referenced previously, consideration of the *digital divide* is crucial to ensure that those who may not have easy access to technology, or the skills required to use it, are not excluded from receiving mental health care delivered via HITs. Recommendations to bridge the digital divide include (1) technology subsidies for low-income consumers, (2) user-friendly technologies appropriate for consumers with physical disabilities and cognitive impairment; and (3) demonstrations and training opportunities for consumers who might not otherwise have the opportunity to learn how to use available technologies [55].

Furthermore, health services are also encouraged to consider the addition of a digital navigator to their care team to improve the uptake and implementation of HITs within care [56]. The role of a digital navigator is 3-fold: (1) evaluate HITs, such as apps, and make appropriate recommendations to health professionals; (2) set up technology and troubleshoot with the consumer, thereby allowing the health professional to focus on the clinical interaction with the consumer; and (3) interpret and report salient data collected by the HIT to both the consumer and the health professional in a user-friendly way to inform care and self-management. Although the current use of HITs among older adults is relatively low, this does not appear to be due to lack of interest [20] but rather due to the need for education and training in relation to the potential benefits of HITs and the practicalities of engagement with these technologies [57]. As such, a digital navigator has the potential to be particularly impactful for the older adult community, including both for consumers and their families and for health professionals.

### Prototyping the InnoWell Platform for Older Adults

Importantly, many of the features and functions suggested by participants for the digital tool align with the core principles underpinning the design and development of the InnoWell Platform, which include increasing access to standardized, broad-based assessment; identifying and tracking consumer needs; matching those needs with personalized care options; and enhancing the quality of care provided to consumers [34]. Although the assessment was not discussed per se, participants recognized that the more information that was input into the digital tool, including via interoperable devices, increased the likelihood of personalized feedback and recommendations. Furthermore, the ability to track and store data over time was valued by participants as a means to better understand what information, resources, and intervention strategies were associated with positive health outcomes based on personal goals relative to those that were not effective for the consumer. It was also recognized that a personal data record, only shared with health professionals with the consumer’s permission, had the potential to facilitate coordinated care across health professionals and services and to prevent the need to retell one’s story repeatedly to new providers. Although it was noted that HITs have the potential to improve access to services, particularly for consumers in regional or remote areas, there was consensus that HITs cannot and should not replace health professionals. Even when developed and delivered by a credible source, it was believed that HITs are not comparable with in-person care and that the connection with a health professional remains a valued part of seeking and receiving care. That said, participants consistently stated that they would make use of an HIT if asked to do so by a health professional.

Despite the willingness to engage with HITs, data privacy and security concerns were frequently referenced as barriers to use, which aligns with previous user-centered work in this area [58]. This is perhaps not surprising, given the frequency of data breaches globally. For example, the United States Department of Health and Human Services’ Office for Civil Rights breach portal listed 510 health care data breaches of 500 or more records in 2019, reflecting a 196% increase from 2018 [59]. Needless to say, adherence to relevant privacy policies is paramount in the development and implementation of HITs to protect consumers’ health information from being disclosed for marketing purposes or, perhaps more importantly, identity theft and fraud.

Importantly, the results of this study have translated to a configuration of the InnoWell Platform specifically tailored to older adults. The broad-based assessment, for example, has been modified to reflect areas of health that are particularly relevant to older adults, including cognition and pain, and to incorporate assessment tools specifically designed for the older adult community (as opposed to tools used in configurations of the InnoWell Platform designed for young people or veterans). All informational materials provided within the InnoWell Platform are appropriate for older adults. For example, fact sheets are provided regarding the benefits of physical activity or the health impacts of excessive alcohol use for older adults. Furthermore, the care options embedded within the InnoWell Platform have been revised to reflect the needs of older adults, such as recommendations for apps for cognitive training, medication management, and cardiovascular health. The design and development of additional features and functionality of the InnoWell Platform based on the information gathered in this study are currently under consideration for inclusion in the next iteration of this innovative HIT.

### Limitations

This study has some limitations that are important to note. All participants in this study were regular users of technology with high levels of digital literacy. As such, the accessibility, engagement, and appropriateness of technology for novice users or those who do not have easy access to technology could not be explored. In addition, only 2 supportive others were included in the participant sample, thereby limiting any conclusions that can be drawn about the features or functions of HITs that may be appropriate specifically for this user group. Finally, to promote patient privacy, no demographic information was collected from participants; therefore, we were unable to comment on factors such as age range, highest level of education, or occupational status (eg, retired). This also precludes the ability to investigate differences in technology preferences based on age (eg*, 50-64 years vs* 65-80 years).

### Conclusions

Older adults readily engage with a range of technologies in day-to-day life, with current participants endorsing a preference for smartphones and computers relative to other devices. HITs have the potential to improve access to cost-effective and low-intensity interventions at scale to improve and maintain mental health and well-being. Participants referenced personalization and the ability to access up-to-date, credible information and resources as primary facilitators of HIT adoption, with a strong desire for integration with standard care practices to preserve personal connections with health professionals. Data privacy and security risks were a primary barrier to HIT use, although this may be mitigated if the source of the digital tool is reputable. Variability in digital literacy among older adults also has the potential to limit the adoption of such tools. However, several strategies may improve uptake and efficacy, including active co-design of HITs specifically with the older adult community to ensure usability, acceptability, and appropriateness; support for HIT selection and use of clinical applications via a digital navigator; and education and training materials embedded within the HIT.

### Future Directions

The configuration of the InnoWell Platform specific for older adults is now being trialed in a naturalistic 90-day user testing study. Participants aged 50 years and older are invited to engage with the InnoWell Platform for a period of 90 days and asked to complete short web-based surveys at 5 time points (baseline [or day 1], day 15, day 30, day 60, and day 90), regarding the quality, usability, and acceptability of the functionality of the prototyped InnoWell Platform. Eighteen participants have enrolled in this study to date, and results are expected to be submitted for publication in early 2021. The findings will inform the iterative redesign and development of the InnoWell Platform before the implementation within an older adult health service setting. Furthermore, participant feedback will also be used in the design and development of other HITs for the older adult community, such as gamified cognitive tests to assess and monitor cognitive functioning over time and multifaceted, interactive web-based interventions to support and maintain mental health and well-being.

## Appendix

Multimedia Appendix 1 Older adult participatory design agenda.

## Abbreviations

BMC: Brain and Mind Centre
HIT: health information technology
