# Supplementary material for: Understanding Technology Preferences and Requirements for Health Information Technologies Designed to Improve and Maintain the Mental Health and Well-Being of Older Adults: Participatory Design Study
Source: JMIR Aging. 2021 Jan 6;4(1):e21461. doi: 10.2196/21461 (PMC7817357; doi:10.2196/21461)
Supplement: Multimedia Appendix 1 [file aging_v4i1e21461_app1.docx]

# **Older Adult Participatory Design Agenda**

**Date:**

**Time:**

**Location:**

**Facilitators:**

**Scribes:**

**Counselling/ Clinician Support:**

**Topic focus**

- Broad discovery
- Envisioning tech for health and wellbeing for older adults

**Materials required**

- Name tags
- Participant Information Sheets
- Participant Consent Forms
- Pens or pencils
- Sharpies
- White board markers
- Post-it notes
- A3 paper
- Blank wireframes
- Tape

**AGENDA ITEMS**

**Introduction (1:00-1:15)**

- Informed consent
- Introductions and housekeeping
- Overview of workshop
- Icebreaker exercise –
  - Name and personal ‘fun’ fact.
  - What is your favourite piece of technology and why?

**Discovery – Understanding technology use among older adults (1:15-2:00)**

- How comfortable are you using technology?
- If you use the internet, what sort of websites/services do you like to use? Do you use any apps or etools?
- Which websites do you avoid? What turns you off when you look at a website – movement, colour, too much text, etc?
- How much time on average do you spend using technology on a typical day?
- Do you use technology to support your health and wellbeing? Have you ever engaged in any online programs related to health (e.g., answered survey questions about your risk of a health related problem, online health interventions, etc.)? What about online IQ tests, brain training, health/nutrition, exercise, etc?
- What sorts of services would you like to see available on the internet?
- What makes you go back to a website – engagement strategies.
- What scares you the most about technology? What excites you most about technology?
- Do you share information with friends, family or doctors via technology?

**Afternoon tea (2:00-2:15)**

**Prototyping: Envisioning tech for health and wellbeing (2:15-3:00)**

- *As a whole Group – using white board*
- If there were no limitations, and anything were possible, what would you want from a technology-solution to support mental health and wellbeing/healthy ageing?
  - Consider: what kind of technology is it? App, website? How do you want to access it – phone, tablet, computer? What could it look like? Who is it for? When would they use it? What does it include? What sort of supports would be provided (ie, information? Cognitive training? Links to doctors?) What about sharing data between apps/websites (ie, integrating wellbeing questionnaires with you fitbit data?)
  - How can technology further support the journey/role individuals, families and clinicians to support mental health and wellbeing for an ageing community?
- Do you have any concerns in providing personal information over an app or website? What makes you trust a website (ie, logos, research evidence, testimonials)? Do you worry about the security of your health information online?

**Individual Journey (3:00-3:30)**

- *Split into smaller groups*
- Using the prototype created, explore how a hypothetical older adult might use the features of this technology.
- For example, create a hypothetical individual (lived experience/supportive other/health professional), their needs, and how they might use the technology.

**Evaluate: Discussion (20 min)**

- Share and discuss each journey
- What other considerations need to be accounted for the older adult community?
- What would make you feel comfortable and safe to access these modes of support online? What would you see as some issues accessing this type of support online?

**Wrap up & thoughts (10 min)**

- Questions
- Thoughts / Feedback
